# Innovative Mobile Manipulator Solution for Modern Flexible Manufacturing Processes

**DOI:** 10.3390/s19245414

**Published:** 2019-12-09

**Authors:** Jose Luis Outón, Iván Villaverde, Héctor Herrero, Urko Esnaola, Basilio Sierra

**Affiliations:** 1 Tecnalia Research and Innovation, Industry and Transport Division, 20009 San Sebastián, Spain; ivan.villaverde@tecnalia.com (I.V.); hector.herrero@tecnalia.com (H.H.); urko.esnaola@tecnalia.com (U.E.); 2 Robotics and Autonomous Systems Group, Universidad del País Vasco/Euskal Herriko Unibertsitatea, 20009 San Sebastián, Spain; b.sierra@ehu.eus

**Keywords:** industrial mobile manipulator, robotics, perception, sensor fusion, autonomous navigation, skill-based programming, Industry 4.0

## Abstract

There is a paradigm shift in current manufacturing needs that is causing a change from the current mass-production-based approach to a mass customization approach where production volumes are smaller and more variable. Current processes are very adapted to the previous paradigm and lack the required flexibility to adapt to the new production needs. To solve this problem, an innovative industrial mobile manipulator is presented. The robot is equipped with a variety of sensors that allow it to perceive its surroundings and perform complex tasks in dynamic environments. Following the current needs of the industry, the robot is capable of autonomous navigation, safely avoiding obstacles. It is flexible enough to be able to perform a wide variety of tasks, being the change between tasks done easily thanks to skills-based programming and the ability to change tools autonomously. In addition, its security systems allow it to share the workspace with human operators. This prototype has been developed as part of THOMAS European project, and it has been tested and demonstrated in real-world manufacturing use cases.

## 1. Introduction

The manufacturing industry is changing. Many traditional industrial sectors have been based on the serial production line paradigm for decades. By constantly evolving their industrial processes to optimize results and be increasingly efficient, they have achieved a highly efficient manufacturing of products, but always based in the manufacturing of large batches of identical products.

In recent years, however, there is a significant shift in market needs [1]. Product personalization and differentiation have become a key factor when purchasing a wide variety of nonbasic products. The paradigm shift is evident in multiple markets, such as cars or electronics, resulting in switching manufacturing processes from *Low Mix/High Volume* to *High Mix/Low Volume* productions. The adaptation to this new production paradigm, recently known as “*mass customization*” [2], is key to keeping the manufacturing companies’ competitiveness in these sectors. Further, other industries with low throughput but high product variability, such as aeronautics, can greatly benefit from this paradigm shift.

Traditional robotics with its programmable automatons of great repeatability does not respond to the current market demand for changing products with small production batches [3]. The robustness and efficiency of the serial production model is highly compromised by the need to perform changes in production equipment, which lacks the cognitive capabilities to support multiple operations in a dynamic environment [4]. This is due to the non-adapatability of traditional robots and the high cost of changing a task to a different one. Very invasive changes in the area and a lot of reprogramming time by qualified specialists are required. This remains expensive due to limited access to skilled operators caused by an aging workforce and faster technology development, even with recent advances in education [5]. This requires new solutions to assist operators and provide collaborative work environments [6].

In any case, the latest reports reveal that at least 85% of the production tasks in major industries (computers and electronics; electrical equipment, appliances and components; transportation equipment; machinery) are automatable involving assembly/tending of machines which are highly repetitive [7]. Thus, even in the case of mass customization, automation and smart scheduling of production lines [8] are the keys to efficient manufacturing.

In order to adapt to new market demands and the needs of modern industrial processes, a new industrial robot has been designed and manufactured. The robot can be classified as an autonomous industrial mobile manipulator (AIMM), since it meets all the criteria established in [9] where different types of mobile manipulators are presented. This kind of robots are currently an important trend in research, with several recent commercial and research examples available [10,11,12,13]. Different AIMMs have been designed and manufactured for a variety of purposes, including tasks such as polishing, sanding, painting, assembly, packaging, logistics, and other challenges of modern industrial processes.

It is equipped with a large number of built-in sensors which give the robot the ability to perceive its surroundings as well as greater autonomy and adaptability. In addition to traditional safety sensors (i.e., safety lasers scanners), a range of complementary ones have been carefully chosen in order to provide the robot with the ability to work together with people safely and without barriers [14].

As it is a mobile manipulator, this new robot has the ability to navigate autonomously through the work cell thanks to its powerful motorwheels and navigation software solutions. Current navigation techniques are very mature approaches but do not respond to all the needs of modern industry. Infrastructure based navigation systems successfully used in industrial environments—e.g., automatic guided vehicles (AGVs)—lack the necessary flexibility and require a high initial investment. For this reason, more and more approaches are being used in the industry based on 2D navigation techniques for static environments, together with the already well-known intelligent path planning techniques [15], dynamic obstacle avoidance [16], and location in the environment [17]. However, these solutions, on many occasions, are not accurate enough for low tolerance operations common in manufacturing, such as part assembly or picking. Approaches using 3D mapping have been introduced to improve robustness in infrastructure-less navigation, using truncated signed distance functions in voxel maps [18] or multiresolution surfel maps [19]. A final accurate approach or docking is thus necessary to complement standard navigation techniques and achieve adequate positioning accuracy.

As a novelty in current mobile manipulators [20], the presented robot incorporates a torso and two arms that allow it to execute more complex tasks and support a greater payload. The control is a software layer based on the robot operating system (ROS) framework [21,22] that eases programming. ROS is a widespread and mature robotic middleware with a large base community of developers, and it is the de facto standard robotics software in the research community.

**Figure 1:**
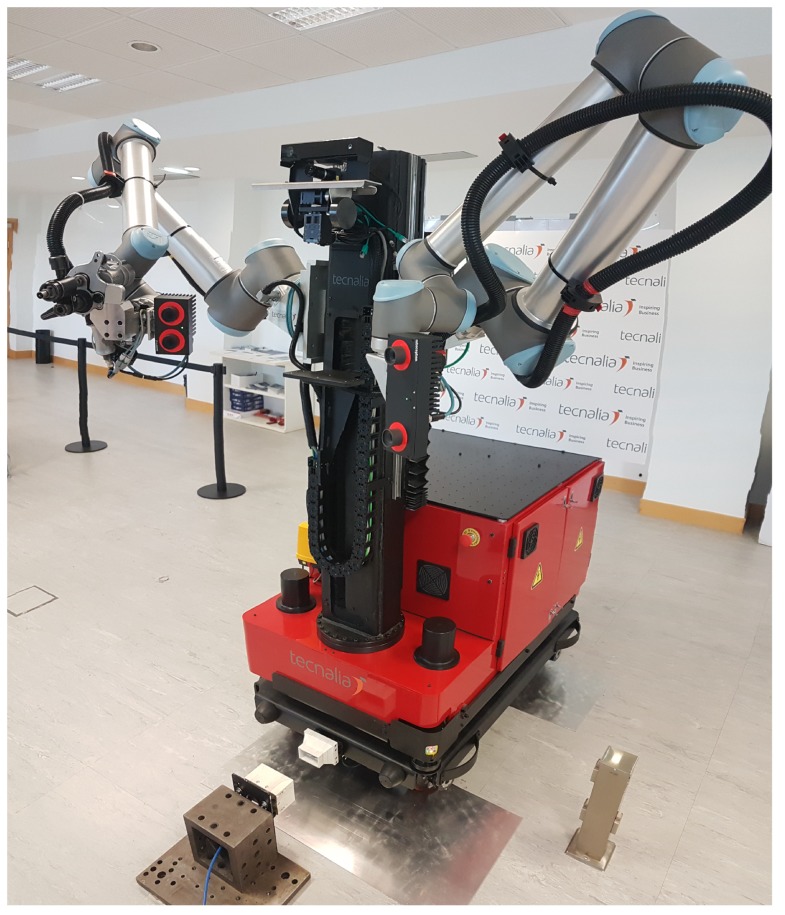
The Mobile Robotic Platform (MRP) navigating autonomously to the charging station.

This AIMM has been carefully designed to be able to address as many of the potential tasks that currently take place in manufacturing processes as possible. The capabilities of this AIMM are being explored as part of the ongoing EU project THOMAS [23], where it is known as the Mobile Robotic Platform (MRP) (Figure 1). This project, with a planned duration of four years, is a framework of collaboration for a consortium of both industrial and R&D partners. The aim of the project is to assess a variety of technological aspects of a new generation of manufacturing systems adapted to the flexibility of the mass customization paradigm (e.g., perception, safety, human–robot interaction, and resource management). The technologies developed by the different partners will be integrated into an industrial robotics solution and tested in two use cases from industrial partners in the automotive (PSA) and aeronautics (Aernnova) sectors. These use cases have enough variety in tasks and operations to demonstrate the versatility of the approach [24].

This paper is devoted to providing a detailed description of the components and systems of the proposed MRP’s prototype. In the following subsections, the project objectives (Section 1.1) and the automotive and aeronautic use cases (Section 1.2) are described. In Section 2, the technical description of the robot is presented. It includes the drive system selected for mobility, a description about the manipulators equipped, and details about sensors and safety components to improve the perception and the autonomy of the robot. Section 3 reviews current navigation techniques and how we use and enhance these techniques to add new capabilities to the robot. These capabilities allow it to reach positions accurately and to improve the cycle times of processes through the ability to work during robot movement. Section 4 describes the skills-based programming that has been applied to our development. Finally, in the last sections, several tests of the system and their results are presented and discussed (Section 5). The paper ends with some conclusions and the following steps in the system development (Section 6).

### 1.1. Objectives

The fourth industrial revolution, called Industry 4.0, demands certain requirements that cutting-edge companies must achieve to attain a satisfactory degree of efficiency [25]. The industry demands have been analyzed, resulting in a proposal of a range of solutions that the presented 4.0 sensorized robotic solution approach is able to offer.

Table 1 shows the needs of the industry for the selected use cases and the objectives that our solution tries to achieve.

### 1.2. Use Cases

#### 1.2.1. Automotive Use Case

PSA is the car manufacturer of two world-famous brands, Peugeot and Citroën. In 2014, it was the second largest European automotive manufacturer and the 9th largest in the world measured by unit production. PSA is an active partner of THOMAS, providing to the consortium open access to the production line of the Mulhouse plant that is being considered under this project.

After a thorough study of the possible applications where the robot could offer a better response and be more autonomous and efficient [26], a complex task was identified that supposes a new technological challenge and of great applicability in the industry.

**Figure 2:**
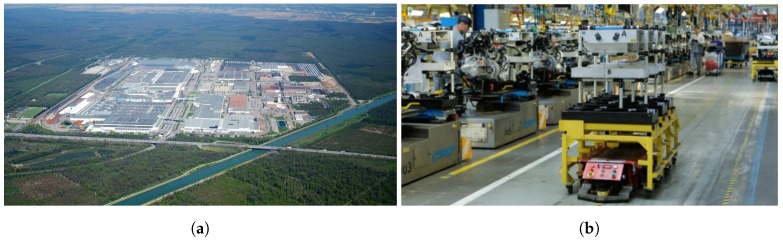
PSA facilities. (**a**) An aerial view of the plant. (**b**) One of the automatic guided vehicles (AGVs) of the plant transporting assembly pieces. ©PSA.

The process is based on the assembly of vehicle damper through a manual process in which several operators interact within an assembly chain. As it is an assembly line, the process is continuous. A filoguided AGV transports the disassembled parts of the damper onto a cart, and at each work station, an operator assembles the parts and leaves the result on the AGV’s cart again, which navigates to the next work station, as can be seen in Figure 2b. This process is cyclic and ends when the damper is fully assembled. Cycle times are critical since the AGV has a predefined trajectory and already established downtime and movement. The maximum efficiency of the process is sought.

In order to save cycle times and take advantage of all the capabilities of the innovative AIMM, it has been proposed to carry out an AGV tracking and following task to perform a threading process of a damper clamping screw. Performing the task in motion greatly reduces the cycle time, since it is not necessary to stop the process, and it is an interesting challenge of manipulation, perception, and control that can help future industrial developments.

The AIMM must navigate autonomously, taking into account obstacles, people, and other unforeseen events from an area of the workshop, where it is performing other tasks, up to the proximity of the AGV. It must also make a previous tool exchange before reaching the AGV. As the autonomous laser-based navigation does not offer very precise results, a precise approximation to the AGV is necessary. Once the AGV starts and begins to move, following the magnetic tape that marks its trajectory, the AIMM must imitate its trajectory in a very precise way so that the robot manipulators can carry out the screw thread threading process. The manner in which the threading is performed and the techniques used are outside the purpose of this article.

#### 1.2.2. Aeronautic Use Case

**Figure 3:**
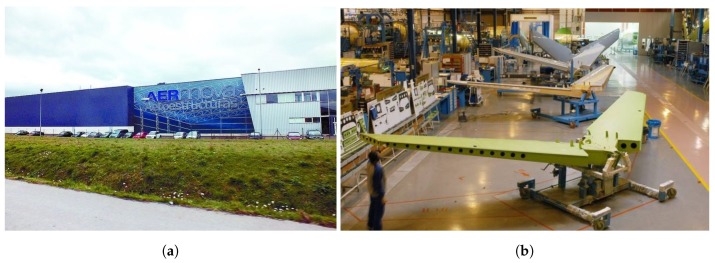
Aernnova facilities. (**a**) Front view of the plant. (**b**) One of the assembly lines. ©Aernnova.

Aernnova Aeroestructuras Alava (SA) is a company dedicated to the assembly of various aircraft structures and is part of the group Aernnova, as can be seen in Figure 3.

Aernnova plant covers an area of 19,900 m${}^{2}$ and has around 600 employees. Currently, the assembly process is mainly manual, having only one automatic machine, CIMPA, for automatic drilling, countersinking, sealing, and riveting metallic skins.

Aernova’s operations have a large number of processes performed manually, many of them with ergonomic problems. Several of them were selected, based on a search for those in which introductions of automation will have more impact in both efficiency and ergonomics.

The main focus was on the drilling process for the joining holes of both skins of a carbon fiber wing to the inner structure (ribs and spar). This drilling is done by means of drilling templates using an automatic drilling unit (ADU). Each template may include 40–70 holes that the robot should drill accurately.

The process presents several challenges which are currently the subject of study by many robotic researchers:

- The robot must acquire the ability to navigate between different workstations autonomously;
- Once inside the workstation, the robot must navigate more accurately to cope with the tight spaces inside the cell and position itself in front of the drilling structure within strict thresholds;
- The robot must be able to change the previously installed tools for the specific ones;
- It should detect all the holes on the template and calculate an obstacle-free trajectory to approach the drill to the structure;
- The perception system must be able to fix a possible incorrect self-referencing of the robot with the structure.

## 2. An Innovative Robot Design

The first and one of the most important phases of building the MRP robotic solution is the correct design of the prototype. Many things have to be taken into account since it is intended to automate as many tasks as possible. The simpler, classical approach of just attaching a robotic arm to a mobile platform, while providing greater versatility than traditional fixed robots, is not enough to cope with the tasks involved in modern industry, especially in the use cases of automotive and aeronautics where the results of this solution is to be validated. As stated previously, recently, many examples have appeared following the AIMM concept of more advanced, industry-ready manipulators [9,10,11,12,13,27]. From a preliminary study of the capabilities of such examples, the benefits and limitations of different systems have been identified, and the MRP has been designed trying to overcome their current limitations and to be able to successfully carrying out the tasks that modern industry is demanding. To do that, the following topics have been taken into account: mobility, manipulation, perception, safety, and autonomy. Table 2 shows a comparison of the main hardware characteristics of the solution proposed in this article (MRP) with other recent mobile manipulators.

### 2.1. Mobility

Mobility is one of the strengths of an AIMM, which is why it needs to be equipped with an adequate traction system.

An AIMM is expected to navigate through typical industrial workshops with flat, smooth grounds and very few ground-level obstacles. Thus, while small irregularities and obstacles should be surmountable, all-terrain capabilities are not required.

For the tasks that are going to be performed, the robot must have great mobility that allows it to reach all the objectives as easily as possible and without having to perform excessive maneuvers. For this reason, cinematic solutions with limited degrees of freedom such as Ackermman, Skid, and differential drives were discarded. Their limited mobility largely conditions the robot’s behavior and ability to move in cluttered environments.

The choice of mecanum wheels [28] was considered initially due to their true holonomic movement capacity. It is a type of wheel widely used among modern mobile manipulators (Kuka KWR iiwa, Robotnik JR2, Clearpath Ridgeback). A robot equipped with them has three complete degrees of freedom, being able to seamlessly move in all directions of the plane, as well as rotate. It was considered a viable option as it provided the highest mobility capacities. However, concerns were raised regarding when combined move/manipulation tasks are performed. As the wheels are composed of small rollers, when they move, they generate vibrations that propagate throughout the robot, increasing its effect farther away from the origin of vibrations. In a robot of these dimensions, small vibrations on the wheels can cause a very significant oscillation in the tip of the arm’s tool center point (TCP). For inspection tasks in motion, application of sealants or paints this system is not recommended.

The final drive configuration chosen for the MRP was a Swerve drive (full 2D drive train in which all wheels are steered) (see Section 3.1.2) composed of four motorwheels, as a good trade between mobility and stability. While not truly holonomic, the motorwheel swerve drive is fully omnidirectional, and the single, medium-sized wheels offer good stability and the capacity of overcome small obstacles and ground irregularities.

The wheels are made of a plastic material that reduces slippage and dampens small pavement damage. The wheels are driven by two large engines, one for translation and one for rotation, the drive system being composed of a total of 8 motors. It reaches a maximum speed of 3 m/s, although it is limited by software to 2 m/s for safety reasons.

The odometry of the robot is provided by the fusion of different sensors. The encoders installed on the wheels provide an approximation of the robot’s odometry. As it is well known, these data are prone to errors (wheel slippage) and drift over time as the error accumulates. An inertial measurement unit (IMU) is also used as additional source of odometry by accumulating over time the provided accelerations data. Finally, a third source of odometry is obtained by matching consecutive laser scans to find the relative translation between acquisition poses [29]. To provide a single, more robust source of odometry, in this robot, we adapted an extended Kalman filter [30] to fuse these three different sources.

### 2.2. Manipulation

In the manufacturing sector, robotic applications are based on fixed automatons that repeat the same task over and over again with great accuracy. A different approach has been sought in our solution, based on flexible mobile robots with the ability to be collaborative. That way, operators can share their workspace with the robot without the need to include barriers.

Many tasks are not solvable through the use of a single robotic arm (i.e., Neobotics MM-700), such as manipulation of large objects, packaging, etc. Dual arm manipulation provides much greater flexibility but requires the ability to simultaneously solve the two arms’ kinematics to achieve synchronous and smooth movements. Based on our previous research in this subject [31], a dual arm system composed of two UR10 collaborative robotic arms was chosen. With a payload of 10 Kg each arm, a combined payload of 20 Kg is achieved, greatly increasing the manipulation capabilities of the system, unlike other double-arm solutions such as the Baxter robot that are limited due to their low payload.

**Figure 4:**
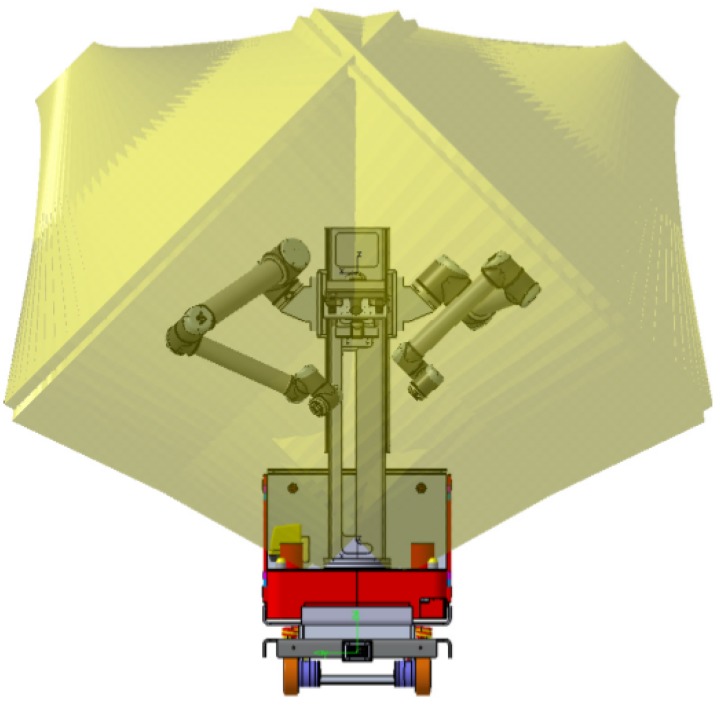
Arms reach volume.

In a dual arm system, the relative position of both arms is a key issue. The relative position between arms must maximize the operational space while minimizing singularities. Thus, a study of the reach of the arms to know the volumes of joint work was done. As a result, it was found that the optimal way to fix the arms to the robot is by using an A-shaped pedestal instead of a V-shaped one (Figure 4).

To provide the robot with greater reachability and therefore increase the useful workspace, arms are mounted over a longitudinal axis in the frontal part of the robot. This vertical axis is also able to rotate, providing two additional degrees of freedom. Rotation covers ±350 degrees of amplitude, allowing the arms to reach objects on the sides and on the back of the robot. The elevation axis has 670 mm of travel. This travel, provided by a threaded spindle, can raise the arms’ base, allowing the TCPs to reach from ground level to a height of 2.5 m.

### 2.3. Components and Systems

Endurance is another key issue in mobile robotics. While static robots are permanently connected to power, mobile robots rely on limited life batteries to operate, which should last enough to not interfere with the production process. Thus, a set of batteries able to work for a whole 8 h turn should be provided. A consumption study of all the electronic devices of the robot showed that a set of lithium batteries of 200 A/h should be enough for normal operation, with the possibility of doing opportunity charging while performing static tasks. Batteries are a big and heavy component, weighing about 80 Kg. They have been placed in the lower part of the robot in order to lower the center of gravity and increase the overall stability of the platform.

A small pneumatic system has also been installed in the MRP. This system is composed of a small compressor and a 5 L air tank. This system is enough to feed pneumatic tools fixed on the robot’s arms and other pneumatic applications with low flow demand, like tool exchangers. For higher demand applications, compressed air can be provided trough a docking mechanism in the frontal part of the platform.

This docking mechanism allows a physical connection between the robot and an external entity. This system is a conical connector where the male part is in the robot and the female part is installed in one or several fixed positions where services (compressed air, power supply) are required by the robot. To avoid proper connection and avoid leaks, both parts must be attached tightly, which requires an accurate positioning of the robot. To successfully achieve this, a vision-based docking system was developed, as described in Section 3.2.1.

In addition to compressed air and power, the docking mechanism allows many other types of services to be passed through, such as digital signals, hydraulic systems, etc.

Flexibility comes from the ability to perform different tasks, which themselves usually require specific or even ad hoc designed tooling. It is, thus, necessary that the robot have the ability to autonomously change tools. The MRP is equipped with a pneumatic claw exchange system, with coupling male/female parts attached to each arms’ last link and each of the tools. The opening and closing of these exchange systems is controlled by a set of solenoid valves, which can be commanded by the controlling software through Modbus.

Since many different tasks and tools are expected to be used, the robot is unable to carry all the required ones. Thus, tools are stored either in a dedicated tool-warehouse area or in the same workspace where the task is to be performed. Thus, the robot needs to detect the position of the required tools when arriving to a specific workspace. A fiducial-tag-based vision system is used to detect the tool stand and perform the tool exchange. Thanks to the skill-based programming system [32], the MRP knows which tool to select in each task.

### 2.4. Perception and Safety

Versatility requires perception of a dynamic environment surrounding the robot. For that, the robot is equipped with a variety of sensors of different nature and purpose. Beyond the internal encoders and IMU, the robot is equipped with:

- Two Sick S300 safety laser scanners mounted on opposite corners of the base;
- Two Roboception rc_visard stereo cameras mounted on the arms;
- One Microsoft Kinect 2.0 mounted at the top of the elevation column;
- One Intel RealSense D435 mounted on the robot arms’ base;
- Two IDS uEye GigE cameras mounted at the front and right side of the base;
- Force torque sensor ATI Delta.

Safety is a critical issue when a shared human–robot workspace is wanted. Thus, the robots must be equipped with adequate safety sensors and measured, in compliance with current regulations. The installed safety lasers cover the entire perimeter of the robot and are attached to a safety rely that breaks the platform and the arms in case of invasion of the defined safety zone. Additionally, the robot has four safety buttons and an additional safety remote that acts the same way.

## 3. Autonomous Navigation

The capacity to autonomously navigate the work environment is one of the main differentiation points of AIMMs with respect to more traditional industrial robotics.

In our approach, two levels of navigation have been identified: long-range, rough navigation (which in a more practical way we call *cell-to-cell* navigation) and short-range, accurate navigation (which we call *in-cell* navigation).

### 3.1. Cell to Cell Navigation

To allow this concept of flexibility in production, it is necessary that robots have the ability to move between different working cells along the workshop, in order to perform as many different tasks as possible. This navigation usually covers relatively long distances in big environments. Further, accuracy in the positioning is not a key issue, usually being in the range of decimeters. Safety and trajectory efficiency are considered more relevant factors.

Laser-based 2D navigation is a mature and well-established technology [33] that is well suited for a traditional industrial workshop. Thus, the approach followed in this paper is that of using state-of-the-art 2D navigation techniques, augmented with the fusion of 3D information provided by additional sensors to cope with some of the limitations of traditional purely 2D approaches. Implementation details are provided in Section 3.1.4 of this paper.

#### 3.1.1. Laser-Based Navigation

The base navigation of the MRP is composed of standard 2D laser-based navigation. Several implementations available as packages of ROS have been configured, tested, and fine-tuned for the mobile robot. This 2D navigation is used as a base system for global and local navigation.

Standard laser-based navigation is typically composed of a two-step approach:

In a first learning step, a simultaneous localization and mapping (SLAM) [34,35] is used to generate a 2D occupancy map. The implementation tested and used is the SLAM approach from [36], available in ROS as the package *hector_mapping*. The main advantage of this approach is that it offers great robustness without depending on additional ego-motion estimation sources (e.g., odometry). One of the key tasks performed for the implementation of the navigation in the MRP has been to fine-tune the algorithm for its specific dynamics.

In the second step, the already learned map is used for navigation. This navigation is also composed of two levels:

- **Localization**: This module provides a position based on the already recorded map. The localization algorithm used in THOMAS is the well-known augmented Monte Carlo localization (AMCL) from [37], available as the popular AMCL ROS package [38]. AMCL is considered as the de facto standard for laser-based localization and is widely used in many applications;
- **Planning**: This module is for both computing safe paths for the robot to travel and send direct speed commands to the drive controller to allow the robot to follow that path. This planning is also made at two levels:
  - **Global planner**: This module is responsible for computing a complete path from the starting point to the goal point. This computation is purely geometric, based on the well-known Dijkstra [39] and A* algorithms. ROS provides implementations for both algorithms in the packages *global_planner* [40] and *navfn* [41], available as plugins of the ROS navigation stack. Both have been tested, *global_planner* being the one normally used;
  - **Local planner**: This module is responsible for two tasks: On the one hand, it computes actual speed commands to follow the planned path, and on the other hand, it adapts the previous path to avoid obstacles in collision course, not present when the original path was computed. There are several packages available in ROS that implement different algorithms and approaches for this task. Three of them have been tested with the MRP, with different outcomes:
    * **dwa_local_planner**: This ROS package [42] implements the classic dynamic window approach (DWA) from [16]. It is a robust and well-proven algorithm. However, the generated trajectories for an omnidirectional robot are somehow very unintuitive for a nonknowing human observer. Since the intended use of the MRP is in collaboration or close by with humans, this approach was abandoned in favor of more modern approaches that allow for better behavior adjustment;
    * **eband_local_planner**: This module [43] uses an elastic band approach, generating bands (curves) that link consecutive points in the path, as described in [44] While obtaining good paths, its current implementation is focused on differential drive robots, so very often, the generated trajectories do not take advantage of the omnidirectional capabilities of the platform;
    * **teb_local_planner**: This package [45] implements the timed elastic band (TEB) approach [46], which tries to optimize the original trajectory with bands that minimize the trajectory execution time, separation from obstacles, and compliance with the kinodynamic constraints. It is the newest approach from the three and allows for greater configuration and fine-tuning of the final behavior. Since the Kinetic ROS version, it implements support (later backported to Indigo version) for omnidirectional robots. A great effort has been dedicated to tuning the planner for the MRP’s specific dynamics. As a result, this by itself allows for safe and robust navigation, as was shown during the Bienal Maquina Herramienta (BIEMH 18) (The Spanish machine tool biennial is the third most important industrial fair in Europe and the first of its sector in Spain. It is held every two years at the Bilbao Exhibition Center (BEC) in Barakaldo, aimed at the main manufacturers, importers, and distributors of machinery and robotics, inviting them to exhibit their products and reach trade agreements with the more than 35,000 buyers in the main countries of the world), where is was part of a demonstrator that was working continuously for 10 h, for 5 consecutive days. However, it still presents some issues in very cluttered environments, in which proximity from obstacles and noise in the sensors cause instability in the generated optimal paths. This, combined with the slow reaction to orientation changes of the MRP due its swerve drive, sometimes results in in very slow speeds.

#### 3.1.2. Low Level Wheel Management

**Figure 5:**
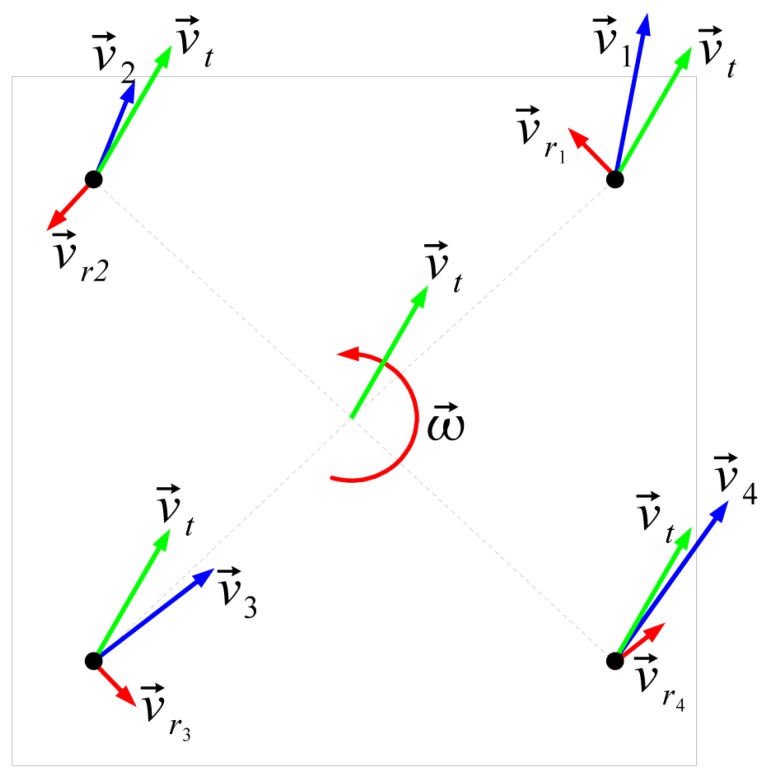
Relationship between each wheel’s speed vector and platform’s speed vector and angular speed in the Swerve drive configuration.

As described previously, the mobile platform is a four-wheeled, omnidirectional platform in the configuration usually referred as “Swerve drive”. The Swerve drive is composed of several (usually four) wheels that can be controlled both in orientation and speed. Each configuration of wheels with their orientation and speed provides a specific linear and angular speed to the center of the platform (Figure 5), providing three degrees of freedom.

The Swerve drive is frequent in omnidirectional platforms since it has several important advantages, such as that it uses simple wheels (that provide better stability) and has greater pushing force (since all wheels provide traction).

However, it also has some important drawbacks, coming mainly from the complexity of its control scheme and build. It has an inherent limitation, since it needs to reconfigure the four wheels to be able to change a robot’s traveling direction. This forces the robot to either stop and reconfigure the wheels or continue while dragging the turning wheels. This problem is more evident in robots with limits in the turning travel of the orientation wheels and with low wheel turning speeds, as is the case with the MRP.

In the case of the industrial application which we tried to automate, a smooth platform movement is a requirement since it combines platform movement with manipulation (e.g., dumper screwing in the automotive use case). Many stop-and-reconfigure motions were not possible due to the nature of the application, as stated. Dragging the wheels was also problematic due to the weight of the platform (putting a lot of stress in the dragging wheels) and the vibrations it creates (propagated to and increased in the arms’ tips).

Thus, the wheel management part of the swerve drive we implemented in the MRP was specifically tuned to adequately balance both possibilities. Three different elements were adjusted:

- Maximize the allowed travel of the orientation wheels, keeping the minimum distance to the physical limits that safety allows;
- Tune the reorienting of the wheel vs. inverting its speed strategy, taking into account the driver’s speeds and acceleration ramps. This management is critical in platform orientation changes, when the new desired orientation of the wheel is far from the current position, as it can be less time costly to invert the traction wheel and orient the wheel at 180${}^{\circ }$ from the original target. The extreme case is when the platform’s traveling speed between moving forward and backward is inverted: It is less time-expensive to not reorient the wheels, but to just invert their traction speed, keeping the orientation;
- Set an acceptable level of dragging (i.e., the maximum difference between target and current wheel orientation to trigger a stop-and-reconfigure action). In the case of the MRP, this difference was set experimentally at 15${}^{\circ }$, as no apparent vibration was propagated to the robot at that dragging level.

Finally, the wheel drivers where also finely tuned to adapt them to the specifics of the MRP and its control system.

#### 3.1.3. Dynamic Robot Footprint

To safely travel the environment, the robot not only needs to know the available space and the position of the obstacles surrounding it, it also needs to be aware of what amount of space itself occupies to know in what positions it can stand in without colliding with any other object in the environment. Traditionally, this space that the robot occupies is known as the robot’s “footprint”.

In 2D navigation approaches, the footprint is estimated as the projection (the plant) of the robot in the map’s plane. A conventional robot usually has a fixed footprint, and current available approaches do not consider the possibility of a robot with a changing form.

This is not the case in a mobile manipulator. This kind of robots are equipped with robotic arms that can project over the base footprint and whose configuration changes over time. Since the navigation system is not aware of this, it creates a situation with high collision risk (e.g., the navigation system tries to go through a door which the base can traverse, but the arms not).

Traditionally, this problem is dealt with by defining a safe travel arm configuration in which arms do not project over the mobile base limits. Every time the robot needs to move, the arms must be put in the travel configuration. This notably increases cycle times and prevents any simultaneous navigation and manipulation.

To avoid that, a new module for dynamic footprint adaptation was developed and is presented in this paper. This module has three functions:

- Monitor the arm’s joints to obtain their position with respect to the robot’s base;
- Compute the bounding polygon of the most external joints and base borders;
- Replace the current footprint with the computed bounding polygon.

Additionally, the standard ROS planner used for navigation has been modified to work with changing footprints, instead of using a fixed one from the start of the execution.

**Figure 6:**
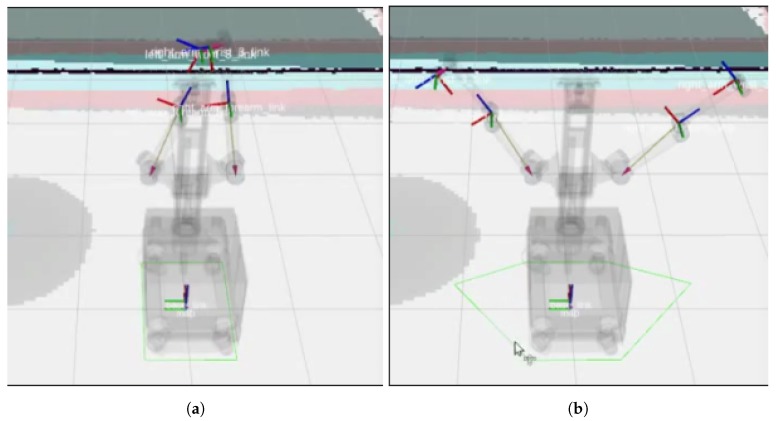
(**a**) Default footprint (green line) covering the MRP’s base. (**b**) Updated footprint covering the stretched arms.

An example of the use of this module can be seen in Figure 6, where in the left there is the “standard” footprint covering the mobile base, while in the right, the footprint has been extended to also cover the stretched arms.

#### 3.1.4. 2D Navigation Improvements by 3D Perception Fusion

As mentioned before, 2D-laser-based navigation is the current state-of-the-art navigation approach for indoor structured environments. The use of the “plant” of the environment as a map is a well-known, widely-used, and robust method for localization and navigation. It also has, however, some well-known limitations, such as its sensitivity to ambiguity in very symmetric environments.

Safety-wise, there is one critical issue that arises by the same nature of the environment representation that the system uses. The “known world” is limited to what the robot’s sensors can see and what can be represented in the map. In the typical laser-based navigation, this is limited to obstacles in a plane at the height at which the laser scanners are mounted. This causes obstacles above and below this plane to be invisible to the robot.

In a typical structured environment, most of the existing obstacles do not pose a threat, since they typically have flat vertical surfaces and go from the ground to some height. Thus, a low-mounted laser can safely detect most of them. However, protruding and hanging obstacles, tables or very low obstacles like pallets still pose a safety threat. One typical and especially dangerous case are the feet, since they can easily project 20–30 cm from the leg (which would be what the robot actually detects) and can be run over even when the robot has detected the person, depending on the robot’s safety configuration.

A full 3D localization/navigation system should be able to overcome these problems. However, the current state of 3D navigation is not so developed and well tested as 2D navigation, so any intent of using such technology will require longer development times with much more uncertain results. Alternatively, standard 2D navigation can greatly benefit from the use of 3D information sources, while retaining its well-known robustness.

##### 3D Perception Source

Multiple possibilities exist for providing 3D information for use in the navigation, both on-board and off-board the MRP. The robot is already equipped with several 3D able cameras, such as the Kinect installed in the torso of the robot and the rc_visard cameras from Roboception. These cameras, however, require a pattern to be projected to be able to get 3D information, so they were discarded for navigation purposes as they would require the projector to be constantly lit.

Alternatively, an Intel RealSense D435 3D camera was mounted on the MRP instead of the Microsoft Kinect due to the fact that the Intel camera has a better resolution and sensor quality.

As the vertical field of view of the camera is limited, how the camera is mounted pn the MRP is relevant to its usefulness for obstacle detection. Basically, three options were possible:

(a) Horizontal mounting: This would be the most standard mounting. However, die to its limited field of view, the camera would only detect obstacles far away from the robot. That will limit its usefulness, since, while the detected obstacles would be used for global navigation, only close obstacles are relevant in the local navigation and are more relevant for safety;
(b) Tilted mounting at low height: A camera mounted this way would be able to detect close-by obstacles at all the height of the robot. However, it has the same problem as the lasers, as it would not be able to detect very low obstacles;
(c) Tilted mounting at high height: It can detect close-by obstacles from its mounting point to the ground. Available mounting points in MRP are high, so it was considered that this mounting would be the one with the greatest obstacle detection capabilities. Its main drawback is the need to filter the ground from the generated point cloud.

**Figure 7:**
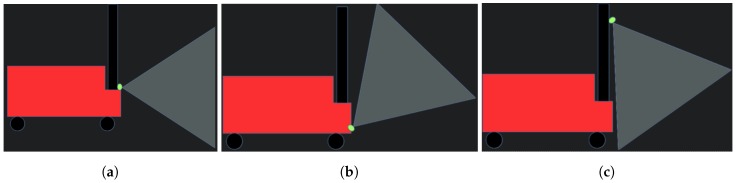
Three different mounting positions of the camera on the robot. (**a**) Horizontal mounting, (**b**) Tilted mounting at low height, (**c**) Tilted mounting at high height.

Final mounting has been done in the auxiliary plate between the two dual arms in the MRP’s torso, pointing down 60${}^{\circ }$ degrees, as shown in Figure 7c. The camera has been manually calibrated so the acquired point cloud has been properly acquired with respect to the robot. To improve performance and reduce noise, the ground has been removed from the point cloud up to a height of 1 cm.

It needs to be considered that a single front-mounted camera has limited functionality in an omnidirectional platform for navigation purposes. It is only able to detect obstacles in front of the robot, whereas the robot can, and usually does, movements backwards and sideways. As it is done with the laser scanners, a fully practical installation requires multiple cameras covering 360${}^{\circ }$ around the robot. On the MRP prototype, only one camera is being used for testing purposes.

##### 3D Obstacle Detection

The 3D information (point cloud) provided by the 3D camera is used for the detection of 3D obstacles.

The point cloud feeds a new layer in the costmap (occupancy grid) used by the navigation. In this layer, instead of using a single, planar cell to represent each map position, a column of voxels is defined. If the position of points in the cloud falls within the voxel, the voxel is marked as occupied. Then, this voxel column is flattened, giving the highest occupation value in the voxel column to the corresponding cell in the navigation map. In this way, any position in the map would be marked as occupied even if the obstacle occupying it is above or below the plane of the laser scanners.

Once the costmap is updated with the 3D sensor information, this information is seamlessly used by the navigation stack, thus allowing the robot to avoid previously invisible obstacles.

**Figure 8:**
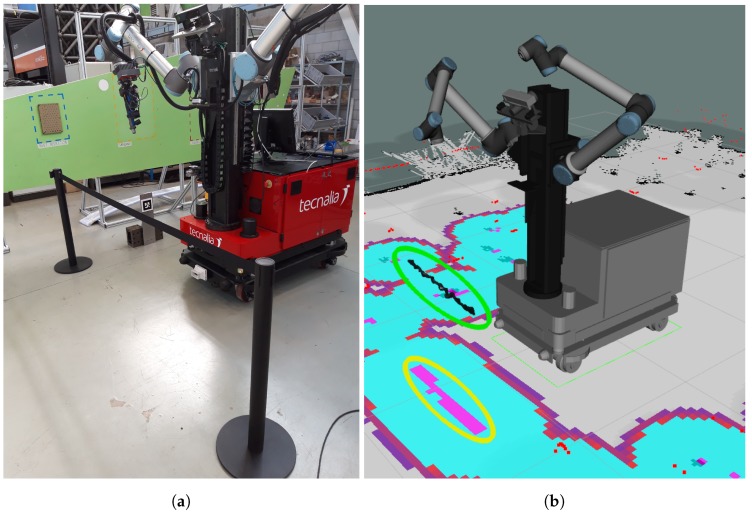
3D obstacle detection. The barrier in front of the MRP (**a**) is detected (highlighted in green in (**b**)) and projected in the obstacle map (highlighted in yellow).

Figure 8 shows and example of how a stanchion barrier, which is an obstacle well above the laser height, is successfully detected and projected to the costmap.

#### 3.1.5. 3D-Perception-Based Navigation

Most commonly used navigation systems rely on laser sensors (e.g., lidar) to calculate the distance to obstacles. As opposed to the classical approach that uses laser sensors as the main input to tackle this task, visual SLAM algorithms use vision sensors to solve the same problems. There are some advantages to using vision sensors in relation to solve the same task with laser sensors:

- Sensor consumption/cost: Vision sensors are typically cheaper than lidar sensors and consume less power, which has an impact on the autonomy of battery-powered mobile robots;
- Re-localization: This relates to both the problem of initialization and the problem of recovering the robot’s localization once the tracking is lost. Vision sensors provide richer information and allow solving this problem in a more efficient way;
- 3D obstacles: Lidar sensors typically provide distance readings in a scanning plane (there are exceptions that provide scanning movements in two axis). This can potentially lead to collisions in case some obstacles cannot be correctly detected;
- Map information: The amount and quality of information provided by a sensor system is much richer, which can be used to produce richer maps.

However, it is important to note that we do not propose to substitute laser sensors with vision. Laser provides very robust readings and allows for safety certification to be achieved, while vision sensors are much harder to certify. We propose to combine both sensor modalities, as it is a good way of improving a system’s robustness and resilience to have redundant information coming from different sources with different parameters of performance and failure modes.

To perform the initial tests, we reviewed the state-of-the-art of visual SLAM methods [47,48], focusing on those with open implementations provided, in order to minimize the integration time required.

After examining more than 20 algorithms and classifying them under different criteria (type of sensor used, type of map created, relocation capability, GPU vs. CPU, and many others), we finally chose the RGBDSLAMv2 algorithm for the initial tests, as it provides very good integration with ROS and has a clean code structure that can be modified. In order to avoid conflicts, we decided to use a Docker-based solution. Docker provides isolation from dependencies in the system, while avoiding any performance penalty as in the case of virtual machines.

**Figure 9:**
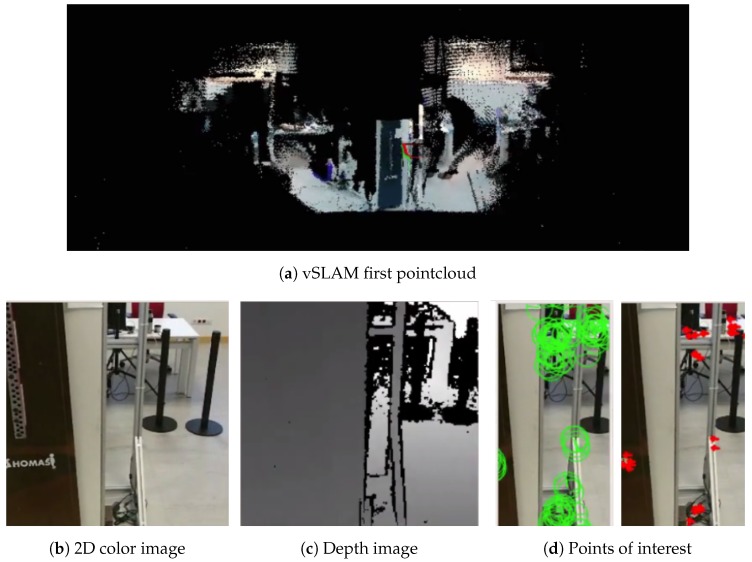
Visual simultaneous localization and mapping (vSLAM) initialization view.

Out of the initial tests performed, Figure 9 shows the initialization of the system. Figure 9a shows the map created, which is represented as a pointcloud. The library offers functions to export the pointcloud to an octomap, which can then be used by other ROS packages to, for example, perform a path planning. Figure 9b shows the initial 2D color image getting from the sensor. In turn, Figure 9c shows the depth obtained thanks to the laser that incorporates the sensor. Figure 9d shows the features located in the 2D image that are used to calculate the camera tracking.

**Figure 10:**
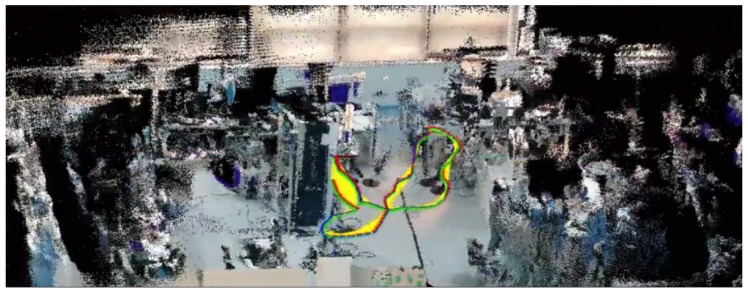
Final 3D map of the environment and robot trajectory.

Figure 10 shows the map produced after a run in the test environment, with the camera/robot trajectory.

### 3.2. In-Cell Navigation

Once in the working cell, an AIMM has to perform a task. Usually, this task is a manipulation that requires high position accuracy to be successful. While standard approaches used in cell-to-cell navigation are good enough for obstacle avoidance and to approximately approach the desired position goal, it is not accurate enough for other tasks. Thus, as a way to achieve this final, accurate positioning, a visual docking mechanism has been developed. Moreover, the use cases include a scenario with a “mobile workstation” in which the MRP needs to perform a screwing operation on the parts that a moving mobile product platform (MPP) is carrying. A mobile “virtual” docking mechanism also has been developed to track and keep a relative position with respect to a mobile goal.

#### 3.2.1. StaTic Docking: Accurate Positioning with Respect to a Static Reference

As described before, the MRP’s internal pneumatic system is only able to provide enough air pressure flow for a few seconds. Thus, it is required that the MRP must attach itself to a docking station that provides enough airflow. This docking procedure required high positioning accuracy, which would also be required by the operations themselves (drilling pattern detection, self-positioning against the wing in sanding processes, etc.).

A fiducial-based visual servoing docking system was developed and tested. The system is based on a proportional control that maintains and ensures, with the desired tolerance, the position of the robot with respect to the marker. To obtain a good response and fluid movements of the robot, a closed loop system with a sampling frequency of 20 Hz is used. Instead of directly using the estimated error in the image, a frame transformation is performed to validate the position and orientation from the detected marker from the camera reference to the base of the robot. This makes the system agnostic of the mount point of the tracking camera.

The position error between both is translated, within a closed loop, as setpoint speed that is sent directly to the robot’s traction system. The system accepts various configuration parameters to achieve precise docking, like maximum speed and goal tolerances. The reference goal was recorded in a previous calibration process. The system allows for both approach (dock) and walk away (undock) from the reference.

**Figure 11:**
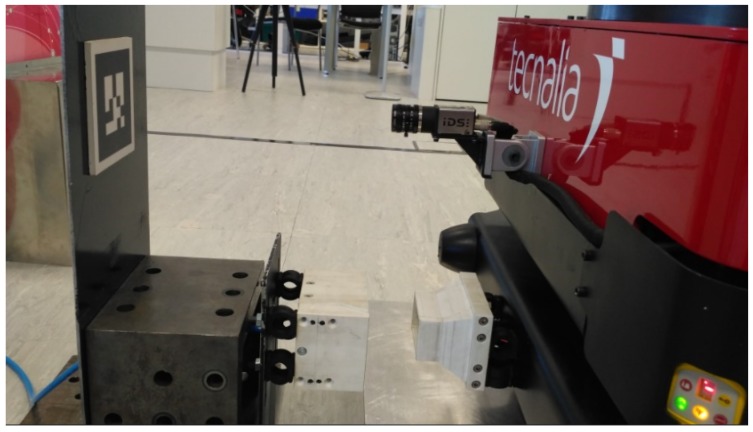
Final docking system with an IDS camera, charge station, and marker installed.

Initial validation of the system was done with the mounted Microsoft Kinect, but is showed some limitations that required a large marker. To solve this, a high-resolution IDS camera was installed in the front of the robot’s base. This improved greatly image quality and accuracy, allowing the use of a much smaller marker, less invasive with the working area. This marker was attached to a docking and charging station able to provide compressed air and power (Figure 11).

#### 3.2.2. Dynamic Docking: Mobile Reference Accurate Positioning and Trajectory Following

In-cell navigation in the automotive use case requires the synchronized navigation of the MRP and MPP. While the developed static docking is robust and accurate, it is not designed to track and follow a moving target. Thus, a different system was required for this task.

##### Perception

**Figure 12:**
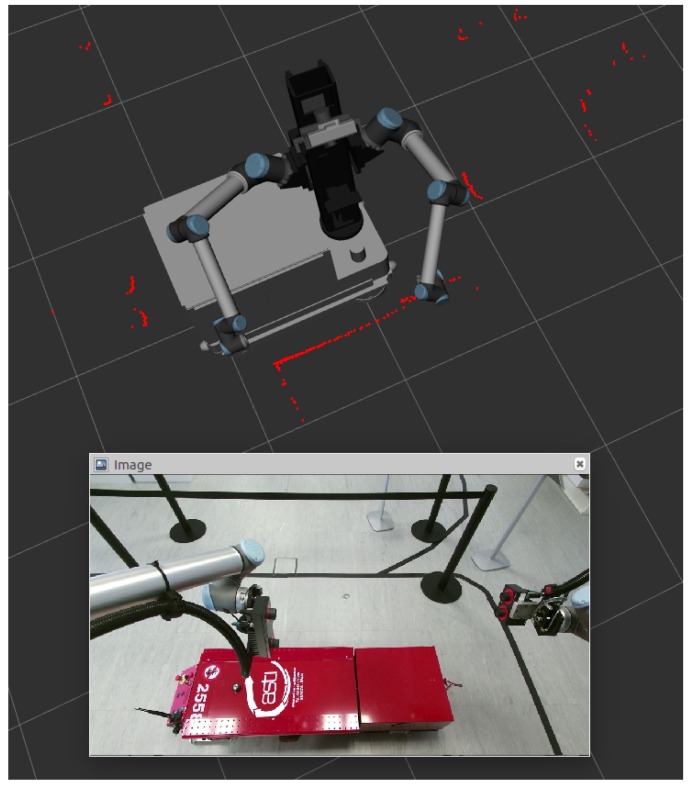
Silhouette of the MPP viewed from the MRP’s laser scanners and correspondent camera image.

Initially, a sensor fusion system using the on-board Microsoft Kinect and the laser scanners was proposed. This approach tried to combine the accuracy of the marker based tracking with the speed of laser scanners. The literature shows many examples of laser based detection and tracking of objects, such as people and vehicles in outdoor conditions [49], or combining SLAM with tracking of moving objects (DTMO) [50]. The basic approach is to match the “shadow” created by the objects in the laser readings [51]. Detection and tracking of the MPP would be done by fusing the information of the lasers with the image coming from the camera, as seen in Figure 12.

Initial tests, however, showed that the head, pan-tilt mounted Kinect camera was not accurate enough for the task, due to the big minimum distance to the target marker, low camera resolution, and difficult calibration of the kinematic chain encompassing the robot’s base, torso and pan-tilt unit.

Similarly to the final configuration of the static docking, an industrial IDS uEye GigE camera was mounted on the robot’s side at low high. This mounting point allowed placing the tracking marker on one of the “legs” of the MPP’s dolly where it both allowed a very close tracking distance (50 cm) and a good position of the torso and arms over the dolly for manipulation tasks.

The fixed mount, high resolution, and close tracking distance provided very good tracking accuracy. Moreover, the camera and tracking system were able to provide up to 30 fps. This speed made the fusion of lasers for increased tracking speed unnecessary.

##### Control

As the goal of the application is to be able to perform some manipulation in the parts carried by the MPP’s dolly, the target of the tracking system is to maintain formation with the MPP. As the union between the MPP and the dolly is not rigid, a steady relative position to the dolly (and not the MPP) should be kept.

While the MPP’s movement is linear in most of its trajectory, the dolly’s movement is more erratic, oscillating depending on different variables, such as the initial position of the wheels or the conditions of the ground.

Thus, control of the MRP should be done in the three possible degrees of freedom: lineal, lateral, and angular speeds. Since the dolly’s movements in each dimension were expected to be very different, each speed component was controlled with its own PID with different gains. This way, for instance, linear PID had much higher reactivity than the lateral or angular ones.

**Figure 13:**
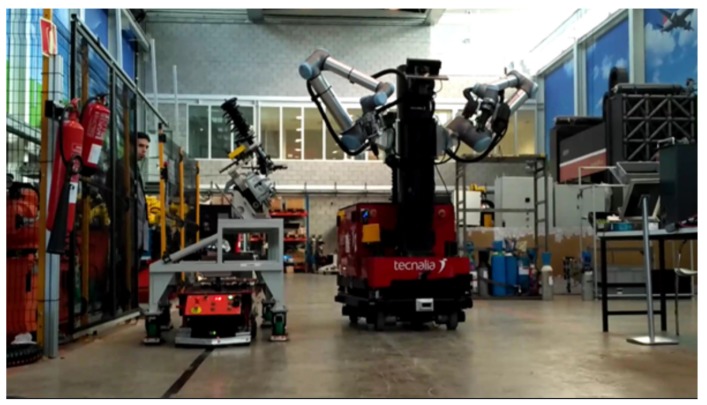
MRP following the mobile product platform (MPP) through the dynamic docking (visual servoing) system.

PIDs were fine-tuned via multiple repetitive testing in our workshop, as can be seen in Figure 13.

The achieved accuracy was in the order of <1 cm once the following stabilizes, which takes approximately two seconds. This error should be mechanically absorbed easily by the arms or tooling.

However, at the start, the error can get as high as 4 cm. This initial high error is inherent to the reactive nature of the feedforward PID and the dynamic reactions of the MRP (command-to-action delays, acceleration ramps, etc.). The initial error can potentially be greatly reduced if the system is able to command both the MPP and MRP to start simultaneously, instead of the MRP to be waiting for the MPP to start moving. Unfortunately, this capacity required some equipment from the AGV provider that was not available at the time of development, but it should be studied in further developments.

## 4. Process Control and Programming

### 4.1. Skill-Based Programming

Skill-based programming has been used in this robotic solution [52]. Each skill represents a singular operation or task (detect one part, move arm to a pose, grasp, etc.) Encapsulated in skills, each task acquires a higher level of abstraction, making it easier to manage the flow of execution and making the system more robust to changes and errors in the processes.

**Figure 14:**
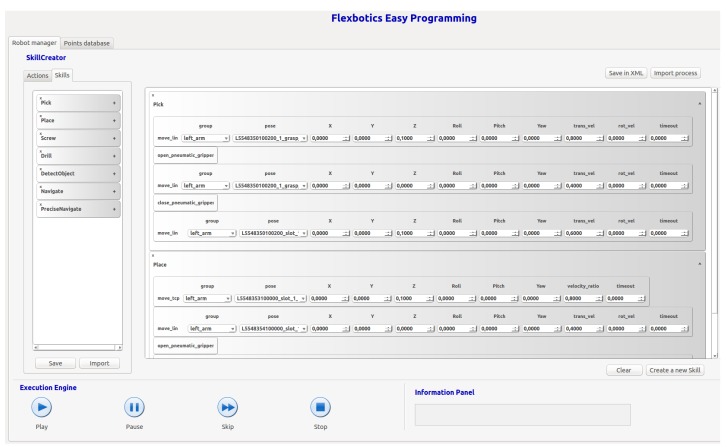
Graphical user interface (GUI) developed for skill management.

Skills can be combined to generate more complex skills to create specific solutions that solve new problems. This skill-based approach reduces the time devoted to programming and allows the reuse of the skill learned in other similar processes. A graphical user interface (GUI) has been developed for the management of skills, as can be seen in Figure 14.

### 4.2. High-Level Task Management and World Model

As part of the THOMAS project, the MRP is integrated into a more complex system ideally composed of several MRPs and a higher-level task management system [53]. A world model also allows for seamless exchange of environment information between different agents (robots, sensors, operators) in the workshop [54].

## 5. Results

The performance of the proposed system was evaluated through a series of tests.

### 5.1. Static Docking

Static docking is used to accurately position the MRP to perform tasks where there is a very low tolerance for a positioning error. Two cases are present in the proposed scenarios: to detect and align against the MPP in the automotive use case and to connect the robot to the pneumatic pressure through the docking mechanism in the aeronautics use case.

A set of tests were performed using the proposed system and without using it (i.e., with the final position given by the cell-to-cell navigation). The number of successes was sought, with a test being considered a success when the final position is accurate enough for the MRP to be able to continue with the next skill in the task. In the case of the Aeronautics, it would be if the external pneumatic system is properly connected, allowing the operation of the ADU. In the the Automotive use case, it would be if the MRP is able to start following the MPP.

A total of 60 tests were carried out within the Tecnalia facilities, which has a mixed illumination of large windows and artificial light.

As can be seen in Table 3, in cases where static docking was used, the robot was able to continue performing its next skill without problem in 96% of the attempts. The recorded failure can be possibly attributable to the reflections of the sunlight on the marker, making the camera unable to detect it.

When not using static docking, in the case of aeronautics, the MRP was unable to insert the docking mechanism in every attempt, thus being unable to activate the ADU. In the case of automotive, the MRP was able to follow the MPP in most of the tests, due to the lesser positioning requirements of the tracking system. However, the initial tracking error was very big, requiring a longer tracking stabilization time and greatly reducing the time available for the screwing operation, making it almost impossible to achieve.

### 5.2. Dynamic Docking

In the case of tracking the MPP through the use of the dynamic docking system, the tests were carried out by performing a prior static docking to guarantee an accurate and repetitive initial tracking position error.

Multiple tests were performed to adjust the parameters of the three PIDs until a valid configuration was obtained for the purpose of screwing on the MPP. Twenty tests were recorded to quantify the values between which the error fluctuates.

**Figure 15:**
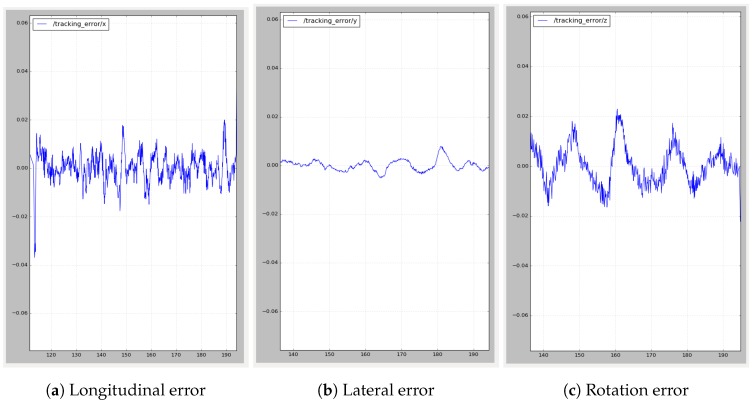
Dynamic docking—tracking error expressed in centimeters between MRP and MPP.

Figure 15 is an example of one of the records, where error in longitudinal (Figure 15a) and lateral (Figure 15b) distances and angular error (Figure 15c) are plotted.

It can be easily appreciated in the left graph that there is a big initial longitudinal tracking error due to the delayed reaction of the MRP when the MPP starts moving. This error is quickly corrected by the dynamic docking system in about 2 s, when the error becomes stationary around ±0.015 m.

**Figure 16:**
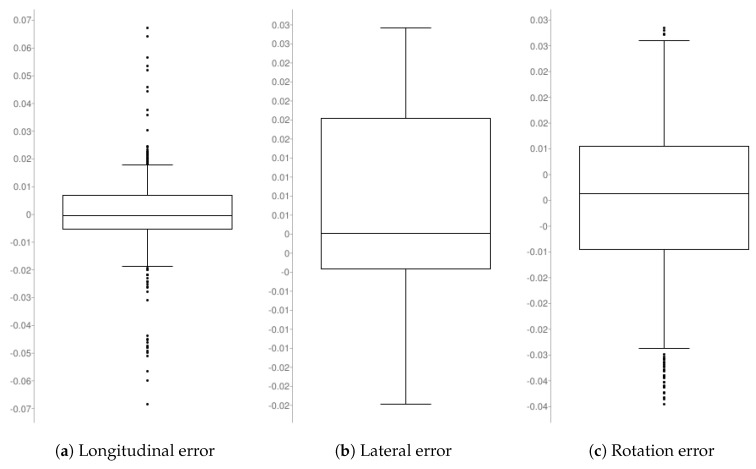
Dynamic docking—results expressed in quartiles.

As can be seen in Table 4 and Figure 16, the maximum and minimum longitudinal errors never exceed 7 cm, corresponding with the mentioned initial error. The first and third quartiles show that most of the time, the error is less than 1 cm, with a mean absolute error of 9 mm. In the case of lateral error, which is not so critical, the mean error is a bit higher. Smooth corrections were prioritized against quickly reducing the error. A relatively high median (that should be very close to zero) shows a bias in the robot’s behavior, keeping it a bit further away from the goal than desired. The angular error is very low overall.

### 5.3. General Results

The docking system proved to be robust, being able to achieve docking successfully from almost any position, given that the marker is in the field of view of the docking camera. The only drawback found was, with it being a vision-based system, a sensitivity to extreme lighting conditions.

The navigation solution was showcased in real live in three demos. A public demonstration was made as part of the Open Doors day organized by the EU Robott-net project [55] in San Sebastian’s Tecnalia premises. The second occasion was the THOMAS integration workshop done at the Laboratory for Manufacturing Systems (LMS), in which the in-cell navigation static and dynamic docking was integrated into the LMS’s second prototype of the MRP. The implemented software system has proven robust when deployed on other robots with different sensors that were tested during development.

Finally, a demonstrator was shown in the BIEMH18 fair, where the MRP was in continuous operation for 5 days, 10 h per day, as mentioned earlier.

Given its robustness, the developed systems are already being used in other projects with AIMM applications with similar needs, like the EU Versatile project [56].

## 6. Conclusions and Future Work

In this paper, we have presented an innovative AIMM design, the MRP used in the project THOMAS. Details of the motivations of the design decision making were provided, as well as the solutions finally adopted.

Several systems of the MRP have been also presented.

The cell-to-cell navigation system is in a good shape, as demonstrated in different tests, such as the BIEMH 18 fair, and 2D navigation was improved through the fusion of 3D sensors information that allows for the detection of obstacles beyond the plane of the laser scanner, improving system safety. The fusion of full 3D navigation is expected to add more robustness in more symmetric and open spaces.

In in-cell navigation, a docking base module was developed. An AR marker was used to self-locate with respect to a precalibrated relative position. The estimated relative position of the marker was translated to control movements. Static docking has shown to be robust and accurate. The big challenge in this area is mobile docking. The performed tests suggest that achieving a reasonably robust and accurate enough mobile docking is envisioned to be possible. However, depending on the nature of operation, the MRP should perform synchronized with the MPP, and it could be necessary to add additional hardware in the arms to physically compensate the remaining error.

The current system navigation capabilities and robustness have been demonstrated in several demos. Further, both static and dynamic docking perform robustly, with only a problem found in extreme lighting conditions.

One of the main identified problems for mobility is the dynamic response of the motorwheels in a complex control scheme like the Swerve drive. As future work, an analytical tool [57] can be used to optimize drive control.

Further developments will be mainly focused on the integration of the systems in the full-scale use of a case demonstrator.

In cell-to-cell navigation, only a few tweaks are expected to be needed, and most development will be devoted to integration of new sources of sensor information (off-board sensors, world model). Full 3D navigation fusion will remain as a less immediate possible improvement.

In in-cell navigation, most of the required work is expected to be done in dynamic docking, trying to improve its accuracy as much as possible to be able to successfully perform the screwing process. 

## Figures and Tables

**Table 1:** Proposed solutions to the industrial needs of the selected use cases.

Industrial Requirements	Objectives
Layout reconfiguration of the production system	Enabling mobility on products and resources by means of mobile resources able to navigate in the shop floor and use dexterous tooling with embedded cognition functions that allow it to perform more than one assembly/logistics operations.
Awareness of real world uncertainty	Enabling perception of the task and the environment using: the individual resource’s sensors to adjust their operation to the real process requirements. collaborative perception by combining sensors of multiple resources and shop floor sensors to collectively plan and execute production tasks.
Adaptation to non-expected situations	Fast programming and automatic execution of multiple tasks. By applying skills over the perceived environment to determine required task adaptation actions and also by automatically generating the robot program for new product variants.
Flexibility to combine different resource types	Safe collaboration between humans and robots eliminating physical barriers(fences, enclosures etc.) by introducing cognitive capabilities that will allow the robots combine different sources to detect the human and its intentions and ensure that no harmful action is taken.

**Table 2:** Comparison of our solution (MRP) with other different recent mobile manipulators.

Robot	Mobility	Manipulation	Perception	Reachability	Autonomy	Robot Payload
MRP	Omnidirectional Swerve drive 3 m/s	(2×) UR10 6 DOF 20 Kg	(2×) Lidar 2D (2×) Stereo camera (2×) HD camera RGB-D sensor IMU Wheel encoder Force Torque sensor	From floor to a 2.5 m Rotation covers 350${}^{\circ }$ of amplitude	>8 h	400 kg
Kuka KMR iiwa	Omnidirectional Mecanum wheels 1 m/s	LBR iiwa 7/14 7 DOF 7/14 Kg	Lidar 2D Arm Force sensor	800–820 mm	-	170 kg
DLR Justin (research)	Omnidirectional Swerve drive 2 m/s	(2×) Kuka LWR 7 DOF 15 Kg	(2×) Stereo camera (4×) RGB-D camera Laser-stripe sensor Torque sensor (2×) IMU	From floor to a 2.7 m	>60 min	20 kg
Neobotics MM-700	Differential 1 m/s	UR10 6 DOF 10 Kg	Lidar 2D	1300 mm	<8 h	180 kg
ClearPath Ridgeback + Baxter	Omnidirectional Mecanum wheels 1.1 m/s	(2×) Custom arms 7 DOF 2/3 Kg	Lidar 2D IMU BumbleBee Stereo cam 360${}^{\circ }$ Sonar sensor	1040 mm + optional pedestal	15 h Only base	100 kg
Robotnik JR2	Omnidirectional Mecanum wheels	AUBO-I5 6 DOF 5 Kg	(2×) Lidar 2D (2×) RGB-D sensor	9245 mm	8 h	100 Kg

**Table 3:** Comparison between the results of using or not using the static docking system.

	SLAM + Static Docking		Only SLAM	
	**Automotive**	**Aeronautic**	**Automotive**	**Aeronautic**
Sunrise	5/5	4/5	4/5	0/5
Noon	5/5	5/5	4/5	0/5
Sunset	5/5	5/5	5/5	0/5
Subtotal	15/15	14/15	13/15	0/15
**Total**	**29/30**		**14/30**	

**Table 4:** Results obtained in the dynamic docking tests between the MRP and the MPP. Error in longitudinal (m), lateral (m), rotation (rad).

	(a) Longitudinal Error	(b) Lateral Error	(c) Rotation Error
Mean (abs)	0.00908	0.01069	0.01232
Median	−0.00041	0.00264	0.00124
Lower value	−0.06834	−0.01981	−0.03949
Higher value	0.06719	0.02957	0.03342
First quartile	−0.00544	−0.00210	−0.00953
Third quartile	0.00675	0.01776	0.01052

## Abbreviations

The following abbreviations are used in this manuscript:

ADU	Automatic Drilling Unit
AGV	Automatic Guided Vehicle
AIMM	Autonomous Industrial Mobile Manipulator
AMCL	Augmented Monte Carlo Localization
BIEMH	Bienal Española de Máquina-Herramienta
DOF	Degree of Freedom
GUI	Graphical User Interface
IMU	Inertial Measurement Unit
LIDAR	Light Detection and Ranging
LMS	Laboratory for Manufacturing Systems and Automation
MPP	Mobile Product Platform
MRP	Mobile Robotic Platform
RGBD	Red Green Blue Depth
ROS	Robot Operating System
SLAM	Simultaneous Localization and Mapping
TCP	Tool Center Point
TEB	Time Elastic Band
